# The effectiveness of using virtual patient educational tools to improve medical students’ clinical reasoning skills: a systematic review

**DOI:** 10.1186/s12909-022-03410-x

**Published:** 2022-05-13

**Authors:** Ruth Plackett, Angelos P. Kassianos, Sophie Mylan, Maria Kambouri, Rosalind Raine, Jessica Sheringham

**Affiliations:** 1 Department of Applied Health Research, UCL, 1-19 Torrington Place, London, WC1E 6BT UK; 2 Department of Global Health and Development, London School of Hygiene and Tropical Medicine, London, England; 3 Institute of Education, UCL, London, UK

**Keywords:** Computer simulation, Virtual patient, Computer-assisted instruction, Educational technology, Medical education, Clinical decision-making, Clinical reasoning, Clinical skills, Review, Medical students

## Abstract

**Background:**

Use of virtual patient educational tools could fill the current gap in the teaching of clinical reasoning skills. However, there is a limited understanding of their effectiveness. The aim of this study was to synthesise the evidence to understand the effectiveness of virtual patient tools aimed at improving undergraduate medical students’ clinical reasoning skills.

**Methods:**

We searched MEDLINE, EMBASE, CINAHL, ERIC, Scopus, Web of Science and PsycINFO from 1990 to January 2022, to identify all experimental articles testing the effectiveness of virtual patient educational tools on medical students’ clinical reasoning skills. Quality of the articles was assessed using an adapted form of the MERSQI and the Newcastle–Ottawa Scale. A narrative synthesis summarised intervention features, how virtual patient tools were evaluated and reported effectiveness.

**Results:**

The search revealed 8,186 articles, with 19 articles meeting the inclusion criteria. Average study quality was moderate (M = 6.5, SD = 2.7), with nearly half not reporting any measurement of validity or reliability for their clinical reasoning outcome measure (8/19, 42%). Eleven articles found a positive effect of virtual patient tools on reasoning (11/19, 58%). Four reported no significant effect and four reported mixed effects (4/19, 21%). Several domains of clinical reasoning were evaluated. Data gathering, ideas about diagnosis and patient management were more often found to improve after virtual patient use (34/47 analyses, 72%) than application of knowledge, flexibility in thinking and problem-solving (3/7 analyses, 43%).

**Conclusions:**

Using virtual patient tools could effectively complement current teaching especially if opportunities for face-to-face teaching or other methods are limited, as there was some evidence that virtual patient educational tools can improve undergraduate medical students’ clinical reasoning skills. Evaluations that measured more case specific clinical reasoning domains, such as data gathering, showed more consistent improvement than general measures like problem-solving. Case specific measures might be more sensitive to change given the context dependent nature of clinical reasoning. Consistent use of validated clinical reasoning measures is needed to enable a meta-analysis to estimate effectiveness.

**Supplementary Information:**

The online version contains supplementary material available at 10.1186/s12909-022-03410-x.

## Background

It has been recommended that more explicit training should be provided in undergraduate medical education on applying clinical reasoning skills, to reduce the impact of future diagnostic errors and potential patient harm [1–4]. Clinical reasoning refers to the thought processes and steps involved in making a clinical judgement [2, 5]. Clinical reasoning requires several complex cognitive skills and is a context dependent skill [2]. It is an evolving and cyclical process that involves applying medical knowledge, gathering necessary information from patients and other sources, interpreting (or reinterpreting) that information and problem formulation (or reformulation) [2, 5]. To be proficient in clinical reasoning, clinicians need to also acquire the requisite knowledge and skills in reflective enquiry [2].

Currently, teaching of clinical reasoning in most medical schools in the UK remains a largely implicit component of small group tutorials, problem-based learning, clinical communication skills sessions, and clinical placements [3]. Making the teaching of these skills more explicit may help students to reflect on their skills, which many models of learning suggest is essential for improving skills [6, 7]. Virtual patient educational tools are becoming increasingly popular in medical education and have been used to explicitly teach clinical reasoning skills [5, 8, 9]. They are defined as “A specific type of computer-based program that simulates real-life clinical scenarios; learners emulate the roles of health care providers to obtain a history, conduct a physical exam, and make diagnostic and therapeutic decisions”. They allow students to practise clinical reasoning with realistic patients, in a safe environment [5, 10]. They may also be particularly suited to providing training on clinical reasoning skills that require deliberate practice with a wide variety and large number of clinical cases. Indeed, many students may have limited contact with patients, where it is also not possible to pre-determine what range of presentations and problems students will meet [5]. Educational and cognitive theories, and empirical research also suggest that virtual patient educational tools could provide an ideal platform for developing clinical reasoning skills if they incorporate best practice features for simulation-based educational tools, in particular providing opportunities for feedback and reflection [6, 7, 10, 11].

Previous systematic reviews and meta-analyses have indicated that virtual patient tools, can significantly improve clinical skills, such as clinical reasoning, for both health professionals and students from a range of disciplines [12–17]. Additionally, reviews have shown that virtual patients used in blended learning have been found to be effective at improving knowledge and skills [15, 18]. However, given that clinical reasoning encompasses several cognitive skills, such as problem-solving and data gathering skills, it would also be useful to understand the impact of virtual patient tools on the different skills or domains of clinical reasoning that were measured, which previous reviews have not explored [12–14, 19, 20]. Furthermore, there has been limited information in previous reviews about whether best practice features for simulation-based educational tools were incorporated into virtual patient tools to improve clinical reasoning [21]. There have also been no sub-group analyses to show the specific effect of these interventions on the clinical reasoning skills of undergraduate medical students, who are likely to have different training needs and ways of learning compared to professionals [12–14].Thus, there is insufficient evidence for undergraduate medical educators to understand the impacts of virtual patient educational tools on the different domains of clinical reasoning for medical students [13, 22]. Medical educators need current information on their effectiveness as the importance and place of online learning in medical education has changed substantially since the COVID-19 pandemic [19, 20]. A timely review is also needed as online learning tools are evolving rapidly and the number of articles evaluating virtual patient tools is increasing year on year [9, 15]. This review, therefore, aims to address the question “How effective are virtual patient educational tools at improving the clinical reasoning skills of undergraduate medical students and which domains of clinical reasoning do they affect?”. Other objectives of this review were to:

- a) identify the use of empirically and theoretically informed intervention features in virtual patient tools, such as reflection;
- b) identify the outcome measures used to assess clinical reasoning skills.

## Methods

This systematic review was conducted following Preferred Reporting Items for Systematic Reviews and Meta-Analyses (PRISMA) guidelines and the PRISMA checklist is available as Additional File 1; the review protocol was presented in RP’s doctoral thesis [23].

### Inclusion and exclusion criteria

Table 1 describes in detail the inclusion and criteria for this review.

**Table 1:** Inclusion and exclusion criteria

Key Concepts	Criteria
Population	Undergraduate medical students Excluded: health professionals, postgraduate students, other health students
Intervention	Interventions that describe an educational method that explicitly teaches clinical reasoning skills and is an interactive computer simulation of real-life clinical scenarios between ‘physicians’ and ‘patients’. The student should emulate the role of a clinician by undertaking various reasoning activities such as gathering data from the patient, interpreting information, or making diagnostic decisions [9]. Patient information could be presented in text or videos on the computer Excluded: high fidelity simulators, manikins, standardised patients, and decision support tools
Comparator	Teaching as usual e.g., no explicit clinical reasoning teaching or a comparison to an alternative method of delivering explicit clinical reasoning teaching e.g., tutorials, problem-based learning discussion groups often involving paper-based instruction Excluded: alternative formats e.g., comparing different types of virtual patient cases
Outcome	Clinical reasoning skills are the thought processes required to identify likely diagnoses, formulate appropriate questions and reach clinical decisions [2]. Interventions that provided sufficient detail to establish whether it improved clinical reasoning skills in a written, oral, or practical test. Commonly used synonyms for clinical reasoning were accepted e.g., clinical decision-making, clinical reasoning, problem-solving, critical thinking, and clinical judgement skills
Study type(s)	RCTs, crossover trials, quasi-experimental studies, and observational studies Excluded: qualitative designs
Publication type(s)	Peer reviewed articles including theses Excluded: conference papers, editorials letters, notes, comments, and meeting abstracts. Articles not in English
Time	Articles from the year 1990, as this was when online learning was beginning to be described [14]

### Search strategy

We applied a search strategy for the following databases: MEDLINE, EMBASE, CINAHL, ERIC, Scopus, Web of Science and PsycINFO, from 1990 to July 2016 and the search was updated to include all articles up to January 2022. Further articles were identified by hand searching the reference lists of included articles. Search terms included a combination of subject headings and key word searches. The full search strategy used in MEDLINE is available as Additional File 2.

### Study selection

One author (RP) screened all the articles retrieved from the search by title and abstract for eligibility of inclusion. Another author (APK) double screened a proportion of the abstracts (736/5,735, 13%,), with moderate agreement (Cohen’s Kappa = 0.64) [24]. The approach taken was that if the first screener (RP) had any doubts, the articles were included for the second screener (APK) to screen. Most ‘disagreements’ were due to APK rejecting those that RP had included but with doubts (29/39, 74% of disagreements) than APK including those that RP rejected (10/39, 26%). Discrepancies were resolved in a consensus meeting and articles were included for full text screening if the abstract lacked enough detail to confirm eligibility. One of the authors (RP) screened all the full text articles and APK double screened a proportion of these articles (60/123, 49%), with moderate agreement (Cohen’s Kappa = 0.65). Discrepancies were resolved in a consensus meeting with the wider team.

### Data extraction

Data on study design, population, setting, delivery of intervention, outcomes, results, and limitations was extracted in an Excel spreadsheet. We also extracted data on the features that were included in the virtual patient tools, such as reflection and feedback. APK and SM piloted the data extraction form with two articles. RP extracted data from 11 articles included in the review, APK extracted data from seven and SM extracted data from one. All extractions were double-checked by either RP, APK and SM; discrepancies were resolved in a consensus meeting.

### Quality assessment

Three authors (RP, APK and SM) assessed the quality of the included articles independently. Quality was assessed using a checklist that incorporated items from two previously developed checklists, the Medical Education Research Study Quality Instrument (MERSQI) and an adapted form of the Newcastle–Ottawa Scale (NOS), which have both been used in previous reviews in this area [14, 22, 25]. The two checklists were incorporated as the NOS was designed to identify aspects of quality related to potential biases in the study design and sample selection, and the MERSQI was designed to identify other aspects of quality, such as the validity and reliability of outcome measures. In addition, articles were given a point if they described how theory informed assessment of clinical reasoning skills or used a previously validated measure that was based on theory e.g., key features problems [26]. Articles could receive a score of up to 14, with scores ranging from 0–4 suggesting low quality, scores of 5–9 suggesting moderate quality and scores of 10–14 indicating high quality.

### Data analyses

We conducted a narrative synthesis of the included articles to address the review objectives. We summarised the characteristics of the interventions to understand what features were included in virtual patient tools and how they were delivered. The study designs used to evaluate the virtual patient tools and the reported effectiveness of each intervention were also reported; Cohen’s *d* effect size was calculated where possible. We also summarised the various clinical reasoning outcome measures used and grouped outcomes measured in each article into specific domains of clinical reasoning informed by the model of clinical reasoning by Higgs et al. [2] and author descriptions of the clinical reasoning outcomes they measured. The analysis of clinical reasoning domains was undertaken at the level of analyses, as articles often reported on more than one domain, and so each domain was included separately in the analysis. In all the articles it was possible to identify at least one domain of clinical reasoning that was measured. Most articles (14/19, 74%) used an aggregate score to represent several domains of clinical reasoning.

## Results

### Study characteristics

**Fig. 1:**
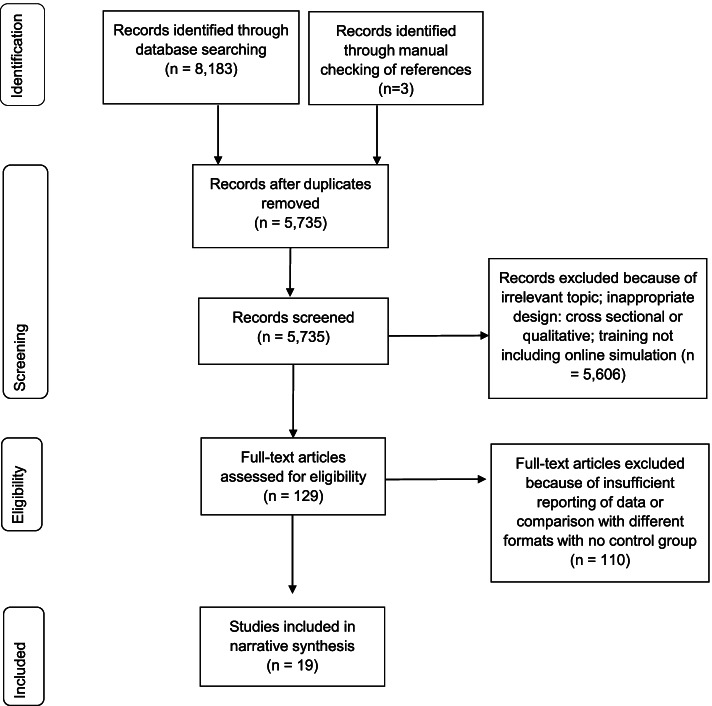
PRISMA (Preferred Reporting Items for Systematic Reviews and Meta-Analyses extension) flow chart for the article search

The search strategy identified 8,186 records of which 19 were included in the review. See Fig. 1 for the PRISMA flow diagram of the number of articles included at each stage of the review. The most common study locations were Germany (7/19, 37%) and the USA (3/19, 16%; see Table 2). Most of the articles were published since 2010 (16/19, 84%).

**Table 2:** Characteristics of the interventions

First author (year)	Country	Virtual Patient tool name	Need to gather data	Delivery	Clinical topic	No. cases	Approximate time to complete one case	Delivered on single or multiple occasions	Feedback used	Reflection used
Aghili et al. 2012	Iran	Not reported	Yes	Solo	Endocrinology	2	Not reported	Multiple	Yes	No
Botezatu et al. 2010	Colombia	Web-SP	No	Solo	Haematology and cardiology	6	1 h	Multiple	Yes	No
Chon et al. 2019	Germany	EMERGE	Yes	Solo	Surgery	4	15 min	Multiple	Yes	No
Dekhtyar et al. 2021	USA	i-Human Patients	Yes	Solo	Abdominal pain and loss of consciousness	3	Not reported	Single	Yes	No
Devitt & Palmer 1998	Australia	MEDICI	No	Solo	Liver disease	5	18 min	Multiple	Yes	No
Isaza-Restrepo et al. 2018	Colombia	The Virtual Patient: Simulator of Clinical Case	Yes	Solo	Gastroenterology	16	2 h	Multiple	Yes	No
Kahl et al. 2010	Germany	Not reported	No	Group	Psychiatry	Not reported	Not reported	Multiple	No	No
Kalet et al. 2007	USA	WISE-MD	No	Solo	Surgery	Not reported	Not reported	Multiple	No	No
Kim et al. 2018	USA	MedU	No	Solo	Multiple	22 (these were required but access to more)	Not reported	Multiple	Yes	No
Kleinert et al. 2015	Germany	ALICE	Yes	Solo	Cancer	3	Not reported	Single	Yes	No
Lehmann et al. 2015	Germany	CAMPUS	No	Solo	Paediatrics	2	1 h	Multiple	Yes	No
Middeke et al. 2018	Germany	EMERGE	Yes	Solo	Accident & emergency	40	9 min	Multiple	Yes	No
Plackett et al. 2020	UK	eCREST	Yes	Solo	Cardio-respiratory	3	13 min	Multiple	Yes	Yes
Qin et al. 2022	China	Not reported	No	Solo	Radiology	5	12 min	Multiple	Yes	No
Raupach et al. 2009	Germany	Clix ®	No	Group	Cardio-respiratory	1	10 h	Multiple	No	No
Raupach et al. 2021	Germany	Not reported	Yes	Solo	Cardio-respiratory	48	11 min	Multiple	Yes	No
Sobocan et al. 2017	Slovenia	MedU	Not reported	Group	Internal medicine	Not reported	Not reported	Multiple	No	No
Watari et al. 2021	Japan	®Body Interact, Coimbra, Portugal	Not reported	Solo	Cardiology and psychiatry	2	20 min	Single	No	No
Wu et al. 2014	China	Not reported	Yes	Solo	Nephrology	4	5 h	Multiple	No	No

### Intervention features

Table 2 describes the characteristics of the interventions. There was a great variety of virtual patient tools that were used to improve reasoning; only two – MedU [27, 28] and EMERGE [29, 30]—were evaluated in more than one study. Just under half of the interventions (9/19, 47%) required the students to gather information from the virtual patient, and were more interactive, while 42% (8/19) were less interactive and presented patients with the patient history already completed. There was not enough information in two articles to determine interactivity (2/19, 11%) [28, 31]. Most of the interventions (16/19, 84%) required students to work individually rather than in groups. Those that were delivered in groups required students to work together to complete the case and make decisions. The clinical topic of the interventions varied; cardiology (5/19, 26%) followed by paediatrics and surgery were the most common topics (2/19, 11% respectively). The number of patient cases within the virtual patient tools ranged from 1–48, with two and three patient cases being the most common number (3/19, 16% respectively). The duration of the patient cases varied from approximately nine minutes to complete a case [32] to 10 h to complete one case (over several weeks) [33]. Most commonly students had multiple opportunities to use and complete the patient cases (16/19, 84%).

Most interventions provided feedback to students on their performance (13/19, 68%). They did this in several ways including: providing the correct answers, providing feedback from experts on how they would have completed the case either via text or video, and discussing answers with a facilitator after completing a case. Reflection was explicitly described in one intervention where users were prompted to reflect during each patient case on their decisions and were required to complete open-ended reflection questions at the end of each case [34]. There were two interventions where the use of reflection was implied, but it was unclear from their description whether the activities were explicitly for reflection [35, 36].

### Study designs and participants

Table 3 describes the characteristics of the included articles including study design, outcome measures used and reported effectiveness. Just under half of the articles were RCTs (9/19, 47%), one was a feasibility RCT (1/19, 5%) [34]. A smaller proportion were non-randomised trials (3/19, 16%) [27, 30, 37] or single group pre-test and post-test design (6/19, 32%). Of those studies with a comparator (*n* = 13), over half of the evaluations (9/13, 69%) compared virtual patient tools to teaching as usual, which included no additional clinical reasoning teaching via any method. In these studies, teaching as usual comprised general clinical teaching via lectures, real patient examinations and small group discussions. Around a third of evaluations (4/13, 31%) compared virtual patient tools directly with an alternative method of explicit clinical reasoning training, which were all tutorials or small group discissions where the same case was discussed [28, 30, 33, 38]. There was a wide variety of year groups that interventions were evaluated with, ranging from those in their 1^st^ year of medical school to those in their 6^th^ year. In most of the evaluations, participants were in their 3^rd^ or 4^th^year of study (8/19, 42% respectively).

**Table 3:** Characteristics of included articles ordered by comparator and study design

Authors and year	Aim(s) of the study	Research Design	Participants—year group, total N and intervention and control group N	Domain of clinical reasoning measured	Outcome measure	Main results	Quality (score out of 14)
**Comparator: teaching as usual**							
Aghili et al. 2012	To evaluate whether virtual patient simulations improve clinical reasoning skills of medical students	RCT	6th years. *N* = 52 (29 IG, 23 CG)	Data gathering, ideas about patient management	Diagnostic test (using patient cases)	⇧ Intervention produced significantly greater improvement in data gathering and ideas about patient management compared to teaching as usual (*d* = 1.55)	Moderate (6)
Botezatu et al. 2010	To explore possible superior retention results with Virtual Patients versus regular learning activities, by measuring the differences between early and delayed assessment results	RCT	4^th^ & 6^th^ years. *N* = 49 (25 IG, 24 CG)	Data gathering, ideas about diagnoses, ideas about patient management	Virtual patient cases	⇧ Intervention produced significantly greater improvement in data gathering, ideas about diagnoses and patient management compared to teaching as usual (average effect size across 5 dimensions, *d* = 1.57)	Moderate (6)
Kahl et al. 2010	To explore whether the addition of systematic training in iterative hypothesis testing may add to the quality of the psychiatry course taught to fifth year medical students	RCT	5^th^ years. *N* = 72 (36 IG, 36 CG)	Ideas about diagnoses	Standardised patient (actor)	⇧ Intervention produced significantly greater improvements in ideas about diagnoses compared to teaching as usual (*d* = 1.17)	Moderate (7)
Kalet et al. 2007	To assess the impact of individual WISE-MD modules on clinical reasoning skills	RCT	Clinical years. *N* = 96 (52 IG, 44 CG)	Data gathering, ideas about patient management	Script concordance test	⇧ Intervention produced significantly greater improvement in data gathering and ideas about patient management compared to teaching as usual (*d* = 0.25)	Moderate (9)
Lehmann et al. 2015	Investigated the effect of Virtual Patients combined with standard simulation-based training on the acquisition of clinical decision-making skills and procedural knowledge, objective skill performance, and self-assessment	RCT	3rd & 4th years. *N* = 57 (30 IG, 27 CG)	Ideas about diagnoses, ideas about patient management, application of knowledge	Key feature problems	⇧ Intervention produced significantly greater improvement in ideas about diagnoses and patient management, and application of knowledge compared to teaching as usual (*d* = 1.91)	High (13)
Qin et al. 2022	To develop a competency-based model of practice-based learning for undergraduate radiology education	RCT	3rd years. *N* = 114 (57 IG, 57 CG)	Application of knowledge	Multiple-choice question examination	⇧ Intervention produced significantly greater improvement in the application of knowledge compared to teaching as usual (*d* = 0.63)	Moderate (5)
Plackett et al. 2020	To assess the feasibility, acceptability and potential effects of eCREST — the electronic Clinical Reasoning Educational Simulation Tool	Feasibility RCT	5th & 6th years. *N* = 264 (137 IG, 127 CG)	Data gathering, flexibility in thinking about diagnoses (reported separately)^a^	Virtual patient case & Diagnostic Thinking Inventory (DTI)	⇧ Ability to gather essential information (data gathering; *d* = 0.19) significantly improved after intervention compared to teaching as usual	High (11)
						⬄ There was no significant difference between groups in relevance of history taking (data gathering; *d* = -0.13) and flexibility in diagnoses (*d* = 0.20)	
Kim et al. 2018	To explore how students use and benefit from virtual patient cases	Non-randomised trial	3^rd^ years. *N* = 255 (129 IG, 126 CG)	Ideas about diagnoses	Clinical rating at end of clerkship by faculty	⬄ Ideas about diagnoses did not significantly improve compared to teaching as usual (voluntary access to cases) (*d* = 0.09)	Moderate (8)
Raupach et al. 2021	To investigate the effectiveness of a digital simulation of an emergency ward regarding appropriate clinical decision-making	Non-randomised trial	4th years. *N* = 100 (58 IG, 42 CG)	Data gathering, ideas about diagnoses, ideas about patient management (reported separately)	Virtual patient cases	⇧ Intervention produced significantly greater improvement in diagnostic accuracy (ideas about diagnoses for 2/3 cases; *d* = 0.81) and patient management (*d* = 0.81), compared to teaching as usual	Moderate (5)
						⬄ Intervention did not significantly improve data gathering, compared to teaching as usual (*d* = 0.03)	
**Comparator: tutorial covering the same case**							
Devitt & Palmer 1998	To evaluate the intervention by assessing whether it expanded students’ knowledge base, improving data-handling abilities and clinical problem-solving skills	RCT	5^th^ years. *N* = 71 (46 IG, 25 CG)	Problem-solving skills	Multi-step clinical problem (patient case)	⬄ Intervention produced non-significantly greater improvement in problem-solving skills compared to tutorial (*d* = 0.50)	Moderate (6)
Raupach et al. 2009	To explore whether students completing a web based collaborative teaching module show higher performance in a test aimed at clinical reasoning skills than students discussing the same clinical case in a traditional teaching session	RCT	4^th^ years. *N* = 143 (72 IG, 71 CG)	Data gathering, ideas about diagnoses, ideas about patient management	Key feature problems	⬄ Intervention did not significantly improve data gathering, ideas about diagnoses and patient management compared to tutorial (*d* = 0.03)	High (10)
Sobocan et al. 2017	To determine the educational effects of substituting p-PBL sessions with VP on undergraduate medical students in their internal medicine course	RCT	3^rd^ years. *N* = 34 (17 IG, 17 CG)	Application of knowledge and flexibility in thinking	DTI	⬄ Intervention did not significantly improve application of knowledge and flexibility in thinking compared to tutorial (*d* = 0.25)	Moderate (7)
Middeke et al. 2018	To compare a Serious Game, the virtual A&E department ‘EMERGE’ to small-group problem-based learning (PBL) regarding student learning outcome on clinical reasoning in the short term	Non-randomised trial	5^th^ years, *N* = 112 (78 IG, 34 CG)	Data gathering, ideas about diagnoses, ideas about patient management (reported separately)	Key feature problems & virtual patient cases	⇧ Intervention produced significantly better clinical reasoning skills compared to tutorial (*d* = 0.47) when measured on key features test and for some domains measured by the virtual patient cases – final diagnosis (ideas about diagnoses),	Moderate (6)
						therapeutic interventions (ideas about patient management), physical examination, instrumental examination (data gathering)	
						⬄ There was no significant difference between groups in history taking (data gathering), laboratory orders and patient transfer (ideas about patient management)	
**Comparator: N/A**							
Chon et al. 2019	To test the effect of a serious game simulating an emergency department (“EMERGE”) on students’ declarative and procedural knowledge	Single group pre & post comparison	Clinical years. *N* = 140	Data gathering, ideas about diagnoses, ideas about patient management, (reported separately)	Patient case	⇧ Diagnostic questions (data gathering; *d* = 0.77), choosing the correct order of diagnostic procedures (ideas about diagnoses; *d* = 0.65) and treatment suggestions (ideas about patient management; *d* = 0.82) improved after using intervention	Moderate (5)
						⬄ There was no significant difference between groups in diagnostic accuracy (ideas about diagnoses; *d* = 0.08)	
Dekhtyar et al. 2021	To test the hypothesis that the Symptom to Diagnosis diagnostic reasoning approach videos paired with practice virtual patient encounter simulations could improve the diagnostic accuracy in medical students as evidenced by their ability to diagnose new simulated cases with diagnoses not previously encountered	Single group pre & post comparison	2nd & 3rd years. *N* = 285	Data gathering, ideas about diagnoses (reported separately)	Virtual patient cases	⇧ History taking efficiency (data gathering; *d* = 0.47), history taking completeness (data gathering *d* = 0.32); efficiency of differential diagnosis (ideas about diagnoses; *d* = 1.16) and completeness of differential diagnosis (ideas about diagnosis; *d* = 0.93) improved after using intervention	Low (3)
Isaza-Restrepo et al. 2018	To present evidence regarding the effectiveness of a low-fidelity simulator: Virtual Patient	Single group pre & post comparison	1st-5th years. *N* = 20	Data gathering, ideas about diagnoses, ideas about patient management	Standardised patient (actor)	⇧ Data gathering, ideas about diagnoses and patient management, and presentation of a case significantly improved after using intervention (average effect size across 5 dimensions from 3 evaluators, *d* = 1.41)	Moderate (6)
Kleinert et al. 2015	To examine whether the use of ALICE has positive impact on clinical reasoning and is a suitable tool for supporting the clinical teacher	Single group pre & post comparison	3rd years. *N* = 62	Ideas about diagnoses, ideas about patient management	Patient cases	⇧ Ideas about diagnoses and patient management significantly improved after using intervention (*d* = 0.92)	Low (3)
Watari et al. 2020	To clarify the effectiveness of VPSs for improving clinical reasoning skills among medical students, and to compare improvements in knowledge or clinical reasoning skills relevant to specific clinical scenarios	Single group pre & post comparison	4th years. *N* = 169	Data gathering, ideas about diagnoses, ideas about patient management	Multiple-choice question quiz (using patient cases)	⇧ Data gathering, ideas about diagnoses and patient management significantly improved after using intervention (*d* = 1.39)	Low (3)
Wu et al. 2014	To examine the effectiveness of a computer-based cognitive representation approach in supporting the learning of clinical reasoning	Single group pre & post comparison	3rd-5th years. *N* = 50	Problem-solving	Concept maps	⇧ Problem-solving significantly improved after using intervention (*d* = 1.17)	Moderate (5)

^a^5 articles reported the impact of the virtual patient tools on each domain of clinical reasoning separately while all others reported an aggregate impact score across several domains of reasoning

### Outcome measures

Six domains of clinical reasoning were identified. Three domains reflected the underlying general cognitive processes required in clinical reasoning and these included: the application of knowledge of the clinical problem derived from theory or experience (3/19, 16%) [28, 39, 40]; flexibility in thinking about diagnoses [28, 34] and problem-solving skills [38, 41](2/19, 11% respectively). One domain reflected more case specific clinical reasoning processes that were measured via data gathering skills, including the relevance of patient examinations (7/19, 37%). Two domains measured the outcomes of the clinical reasoning process in specific cases by measuring the clinical judgements the students made. These included: ideas about diagnoses, including diagnostic accuracy (10/19, 53%), and ideas about patient management, including appropriateness of treatment plans or therapeutic decisions (7/19, 37%).

Under half of the evaluations (8/19, 42%) used measures of clinical reasoning that have been previously reported and validated in the wider literature. These included: key features problems [26, 42](3/19, 16%) [30, 33, 40]; Standardised Patients, where an actor simulates a patient (2/19, 11%) [35, 36]; the Script Concordance Test [43] (1/19, 5%) [44] and the Diagnostic Thinking Inventory [45] (DTI; 2/19, 11%) [28, 34]. In five evaluations (5/19, 26%) student performance was assessed using text-based cases that the authors had developed, often followed by open or multiple choice questions regarding history taking, diagnosis and treatment [29, 31, 38, 46, 47], five used additional virtual patient cases (5/19, 26%) [30, 34, 37, 48, 49], one used a clinical rating by faculty at the end of the students’ clerkship [27], one used a multiple choice examination [39] and one used concept maps (1/19, 5% respectively) to assess five aspects of performance [41].

### Quality of included articles

Additional file 3 gives a detailed breakdown of the quality of the included articles. The average quality was moderate (M = 6.5, SD = 2.7). Only three articles (3/19, 16%) were high quality [33, 34, 40], most were of moderate quality (13/19, 68%) and three were of low quality (3/19, 16%) [31, 47, 49]. Just over half of the articles (10/19, 53%) described how theory informed the evaluation, by either describing theoretical frameworks they used to assess clinical reasoning or using previously developed and validated measures of clinical reasoning. Only four articles (4/19, 21%) reported measuring three or more different types of validity and reliability [33, 34, 40, 50] and nearly half did not report any measurement of validity or reliability (8/19, 42%). Only two (2/19, 11%) articles reported that they selected students from more than one medical school [34, 49]. Three articles (3/19, 16%) reported that the assessor of the outcome was blinded to group allocation. Just over a quarter (5/19, 26%) reported a power calculation, although this was not necessary to calculate for all study designs.

### Reported effectiveness

Just over half of the articles (11/19, 58%) reported that virtual patient tools had significantly positive effects on medical students’ clinical reasoning skills, four articles found no effect [27, 28, 33, 38] and four reported mixed effects (4/19, 21%) [29, 30, 34, 37].

#### Effectiveness by article quality

**Fig. 2:**
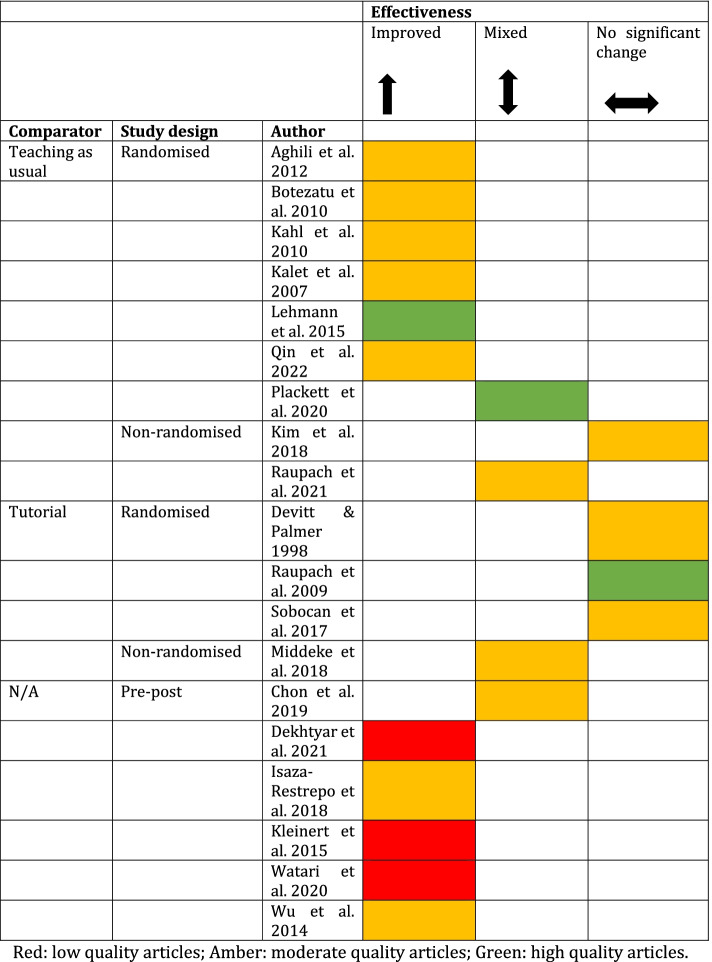
Effectiveness of virtual patient tools by comparator, study design and quality

Of the three articles rated as high-quality, one found no significant effect of virtual patients on reasoning [33], one a positive effect (1/3, 33%) [40], and one a mixed effect [34]. Out of the articles that were rated as moderate quality, most reported virtual patient tools had significant benefits (7/13, 54%) than mixed (3/13, 23%) [29, 30, 37] or neutral effects (3/13, 23%) [27, 28, 38]. The three articles that were rated as low quality all reported virtual patient tools had significant benefits (3/3, 100%; Fig. 2) [31, 47, 49].

#### Effectiveness by study design

Of the articles that used randomised study designs (10/19, 53%), over half (6/10, 60%) reported that virtual patient tools improved clinical reasoning skills compared with controls [36, 39, 40, 44, 46, 48]. Around a third (3/10, 30%) of randomised study designs reported that virtual patient tools had no significant effect [28, 33, 38] and 10% (1/10) found they had mixed effects on clinical reasoning skills compared to controls [34]. Of the articles that used non-randomised trial study designs (3/19, 16%), two found mixed effects of virtual patient tools on clinical reasoning skills compared to controls [30, 37] and one found no significant effects [27]. Of the six articles (6/19, 32%) that used a single group pre and post study design, five articles (5/6, 83%) found a significant improvement in clinical reasoning after using virtual patient tools [31, 35, 41, 47, 49]; only one article (1/6, 17%) reported mixed results (Fig. 2) [29].

#### Effectiveness by comparator

Articles that compared virtual patient tools with teaching as usual (9/19, 47%) reported mostly (6/9, 67%) positive effects on clinical reasoning [36, 39, 40, 46, 48, 50], but two found mixed effects (2/9, 22%) [34, 37] and one found no effect on reasoning (1/9, 11%) [27]. Articles that compared virtual patient tools to tutorials (4/19, 21%) mostly found no effect of virtual patient tools (3/4, 75%) [28, 33, 38] and one showed mixed effects (1/4, 25%) [30] (Fig. 2).

#### Effectiveness by domain of clinical reasoning measured and measurement

**Fig. 3:**
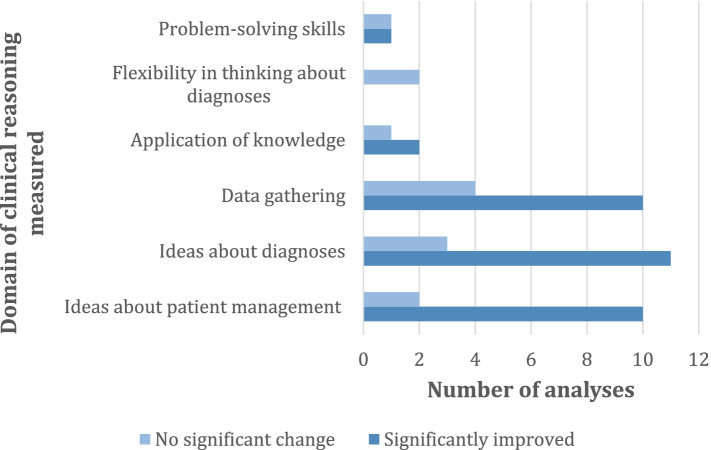
Frequency of analyses that reported different domains of clinical reasoning by effectiveness Note. Total number of analyses = 47 and total number of articles that reported these analyses = 19

Data gathering, ideas about diagnoses and patient management were largely found to significantly improve after virtual patient use (34/47 analyses, 72%; Fig. 3). Application of knowledge, flexibility in thinking about diagnoses and problem-solving skills showed more mixed results, with less than half of these analyses showing significant improvement in these skills (3/7, 43% analyses).

Of the 10 articles that used a patient case (text or virtual) and a bespoke measuring rubric to assess clinical reasoning, over half reported positive effects of using virtual patient tools (6/10, 60%), less than half (4/10, 40%) reported mixed effects [29, 30, 34, 37] and one article reported neutral effects [38]. Half of the articles that used measures of clinical reasoning that have been developed and validated in previous literature, such as the key feature problems, reported significant benefits of using virtual patient tools (4/8, 50%) [35, 36, 40, 44], a quarter reported no significant effects (2/8, 25%) [27, 28, 33] and a quarter reported mixed effects (2/8, 25%) [28, 34].

## Discussion

This review of published evaluations of virtual patient educational tools found there is some evidence that they can improve medical students’ clinical reasoning. Improvements were more consistently reported for domains of clinical reasoning that were more case specific, such as ideas about diagnoses and data gathering, rather than more general reasoning processes, such as problem-solving.

### Intervention features

This review illustrates the diversity in design, content, and delivery of virtual patient tools and the clinical context in which they are applied. Most virtual patient educational tools have been designed for individuals to complete. Many of the tools included features that educational theories and empirical research suggests are important to include in simulation-based learning, such as feedback, but relatively few reported how they facilitated reflection [32, 34–36]. A previous review exploring the impact of virtual patients on communication skills found that the inclusion of a pre-activity with a protocol-informed tutorial, post-activity of debrief or reflection, scaffolding and human feedback improved the effectiveness of the virtual patient tools [21]. Further consideration of how to facilitate reflection and other best practice features in virtual patient tools could allow them to be even more effective at developing reasoning skills [7, 51, 52]. There was also variety in the level of interactivity with the virtual patient tools, with half of the tools not requiring students to gather information from the patient. Previous research is inconclusive as to whether greater interactivity produces better learning outcomes [53]. Studies have shown greater interactivity can facilitate deeper learning and more engagement from users, but it can also increase cognitive load, which can interfere with learning [50, 53]. However, virtual patient tools that allow for greater interactivity might be more helpful for educators to observe and assess clinical reasoning skills, as students can demonstrate a broader range of skills in real-time, such as data gathering.

### Effectiveness

Our results largely concur with previous reviews that have found virtual patient tools are better than no intervention but might not be superior to other methods of explicitly teaching clinical reasoning, such as problem-based learning tutorials [12–15, 17, 18]. The benefits to using virtual patient tools are that they can be used in circumstances when face-to-face teaching is not possible, e.g., due to a pandemic, or because access to patients is limited. Additionally, once upfront costs are covered, the cost of adapting and scaling up can be low. This review suggests that using virtual patient tools can effectively complement face-to-face teaching and as previous reviews have suggested, they could be a particularly useful tool for a blended learning approach to teaching [15, 18]. This review provides useful evidence for medical educators to guide their decisions about using this technology, which may be especially attractive if there is no other explicit teaching of clinical reasoning skills in the curriculum. Further research is needed to understand the context in which different teaching methods are most effective and the feasibility of implementing into curricula, so that medical educators can make more informed decisions on educational methods.

This review showed some evidence that effectiveness might depend on the domains of clinical reasoning that the virtual patient tools were designed to address and how these were measured. Most articles evaluated the effects of virtual patient tools on domains of data gathering, ideas about diagnoses and patient management and many showed significant improvement in these domains. The application of knowledge about clinical problems and processes, flexibility in thinking about diagnoses and problem-solving skills were less commonly measured and showed less consistent improvement after virtual patient use. These findings could be due to issues with measuring different domains of clinical reasoning. Data gathering skills, ideas about diagnoses and patient management are domains that are related to students’ judgements on specific cases. Therefore, they are easier to measure using patient cases and measures like the key feature problems, which are case specific and may be more sensitive to change immediately post intervention. In contrast, the application of knowledge, flexibility in thinking about diagnoses and problem-solving measures may be more related to the underlying cognitive processes of clinical reasoning. These general cognitive skills are less likely to vary over the short-term and measurements, such as the DTI, have not necessarily been designed to be sensitive enough to detect short-term changes in these skills [54, 55]. Case specific outcomes may also be more appropriate for measuring clinical reasoning, as clinical reasoning is a skill that is context dependent [2]. We also found most articles reported aggregated effectiveness over several domains. Future research would benefit from defining the specific domains of clinical reasoning their virtual patient tool aims to improve and provide separate analyses for each aspect. Furthermore, a greater understanding of the psychometric properties of measures of clinical reasoning is needed to identify which domains of reasoning virtual patient tools can effectively teach students and over what timescales.

### Limitations

It was not meaningful to conduct a meta-analysis to summarise the overall effectiveness of virtual patient tools on clinical reasoning due to the substantial heterogeneity in the design and content of the virtual patient tools, the measures of clinical reasoning and the characteristics of samples. Many articles developed their own measures of reasoning but with limited validation it was difficult to ascertain what they were measuring and how comparable they were to other measures. The findings of the review were limited by the lack of high-quality articles that were included. The review was updated in January 2022 and by this time the review authors’ article on a virtual patient tool was eligible for inclusion. This was rated of high quality, and it is possible the authors were biased in their scoring of their own article. As found in previous reviews, most single group pre-test and post-test evaluations found significant benefits of using virtual patient tools and it is possible there was publication bias with negative findings being unpublished [13, 14]. The review was also limited by the small percentage of abstracts that were double screened for inclusion. However, the agreement between screeners was good and any discrepancies were discussed; abstracts where there was uncertainty of inclusion were included in the full text review to ensure we captured as many relevant articles as possible [56].

## Conclusion

Overall, the evidence suggests virtual patient tools could effectively complement current teaching and may be particularly useful if opportunities for face-to-face learning are limited. This research found that evaluations that measured clinical reasoning by measuring case specific domains of clinical reasoning, such as ideas about diagnoses or data gathering, showed more consistent improvement in reasoning than more general measures of reasoning, such as problem-solving. Case specific measures of clinical reasoning may be more sensitive to change following virtual patient cases because they reflect the context dependent nature of clinical reasoning skills. Future evaluations should provide evidence of the validity and reliability of their clinical reasoning outcome measures to aid the comparison of effectiveness between studies. More understanding is needed about how features of virtual patient design and delivery relate to effectiveness.

## Supplementary Information

**Additional file 1.** PRISMA 2020 checklist.

**Additional file 2.** Search history for medline, embase, psychinfo.

**Additional file 3.** Quality assessment of included studies.

## Data Availability

The dataset supporting the conclusions of this article is included within this article and its additional files.

## References

[1] Cleland JA, Abe K, Rethans J-J (2009). The use of simulated patients in medical education: AMEE Guide No 42. Med Teach.

[2] Higgs J, Jones MA, Loftus S, Christensen N. Clinical Reasoning in the Health Professions. UK: Elsevier; 2008.

[3] The Special Interest Group of the Wolfson Research Institute for Health & Wellbeing Durham University. Page G, Matthan J, Silva A, McLaughlin D. Mapping the delivery of ‘Clinical Reasoning’ in UK undergraduate medical curricula. 2016. http://clinical-reasoning.org/resources/pdfs/Mapping-CR-UK-undergrad.pdf. Accessed 3 May 2022.

[4] Io M (2015). Improving Diagnosis in Health Care.

[5] Cook DA, Triola MM (2009). Virtual patients: a critical literature review and proposed next steps. Med Educ.

[6] Ericsson KA (2008). Deliberate practice and acquisition of expert performance: a general overview. Acad Emerg Med.

[7] Kolb DA (1984). Experiential learning : experience as the source of learning and development.

[8] Bradley P (2006). The history of simulation in medical education and possible future directions. Med Educ.

[9] Kononowicz AA, Zary N, Edelbring S, Corral J, Hege I (2015). Virtual patients - what are we talking about? A framework to classify the meanings of the term in healthcare education. BMC Med Educ.

[10] Barry Issenberg S, Mcgaghie WC, Petrusa ER, Lee Gordon D, Scalese RJ (2005). Features and uses of high-fidelity medical simulations that lead to effective learning: a BEME systematic review. Med Teach.

[11] McGaghie WC, Issenberg SB, Petrusa ER, Scalese RJ (2016). Revisiting ‘A critical review of simulation-based medical education research: 2003–2009’. Med Educ.

[12] Consorti F, Mancuso R, Nocioni M, Piccolo A (2012). Efficacy of virtual patients in medical education: A meta-analysis of randomized studies. Comput Educ.

[13] Cook DA, Erwin PJ, Triola MM (2010). Computerized virtual patients in health professions education: a systematic review and meta-analysis. Acad Med.

[14] Cook DA, Levinson AJ, Garside S, Dupras D, Erwin P, Montori V (2008). Internet-based learning in the health professions. J Am Med Assoc.

[15] Kononowicz AA, Woodham LA, Edelbring S, Stathakarou N, Davies D, Saxena N, Tudor Car L, Carlstedt-Duke J, Car J, Zary N (2019). Virtual Patient Simulations in Health Professions Education: Systematic Review and Meta-Analysis by the Digital Health Education Collaboration. J Med Internet Res.

[16] Richardson CL, White S, Chapman S (2020). Virtual patient technology to educate pharmacists and pharmacy students on patient communication: a systematic review. BMJ Simul Technol Enhanc Learning.

[17] Foronda CL, Fernandez-Burgos M, Nadeau C, Kelley CN, Henry MN (2020). Virtual Simulation in Nursing Education: A Systematic Review Spanning 1996 to 2018. Simul Healthc.

[18] Vallée A, Blacher J, Cariou A, Sorbets E (2020). Blended Learning Compared to Traditional Learning in Medical Education: Systematic Review and Meta-Analysis. J Med Internet Res.

[19] Muller DP (2021). Valerie; Amiel, Jonathan; Anand, Shashi; Cassese, Todd; Cunningham, Tara; Kang, Yoon; Nosanchuk, Joshua; Soriano, Rainier; Zbar, Lori; Karani, Reena: Guiding principles for undergraduate medical education in the time of the COVID-19 pandemic. Med Teach.

[20] Hege IS, Sudacka M, Kononowicz AA, Nonnenmann J, Banholzer J, Schelling J, Adler M, Espinoza B, Garrido MA, Radon K (2020). Adaptation of an international virtual patient collection to the COVID-19 pandemic. GMS Journal for Medical Education.

[21] Lee J, Kim H, Kim KH, Jung D, Jowsey T, Webster CS (2020). Effective virtual patient simulators for medical communication training: A systematic review. Med Educ.

[22] Cook DA, Hatala R, Brydges R, Zendejas B, Szostek JH, Wang AT, Erwin PJ, Hamstra SJ (2011). Technology-enhanced simulation for health professions education: a systematic review and meta-analysis. JAMA.

[23] Moher D, Liberati A, Tetzlaff J, Altman DG, The PG (2009). Preferred Reporting Items for Systematic Reviews and Meta-Analyses: The PRISMA Statement. PLoS Med.

[24] McHugh ML (2012). Interrater reliability: the kappa statistic. Biochem Med (Zagreb).

[25] Reed DA, Cook DA, Beckman TJ, Levine RB, Kern DE, Wright SM (2007). Association between funding and quality of published medical education research. JAMA.

[26] Page B (1995). Allen: Developing key-feature problems and examinations to assess clinical decision-making skills. Acad Med.

[27] Kim S, Willett LR, Pan WJ, Afran J, Walker JA, Shea JA (2018). Impact of Required Versus Self-Directed Use of Virtual Patient Cases on Clerkship Performance: A Mixed-Methods Study. Acad Med: J Assoc Am Med Coll.

[28] Sobocan M, Turk N, Dinevski D, Hojs R, Pecovnik Balon B (2017). Problem-based learning in internal medicine: virtual patients or paper-based problems?. Intern Med J.

[29] Chon SH, Timmermann F, Dratsch T, Schuelper N, Plum P, Berlth F, Datta RR, Schramm C, Hander S, Spath MR (2019). Serious Games in Surgical Medical Education: A Virtual Emergency Department as a Tool for Teaching Clinical Reasoning to Medical Students. Jmir Serious Games.

[30] Middeke A, Anders S, Schuelper M, Raupach T, Schuelper N (2018). Training of clinical reasoning with a Serious Game versus small-group problem-based learning: A prospective study. PLoS ONE.

[31] Watari T, Tokuda Y, Owada M, Onigata K (2020). The Utility of Virtual Patient Simulations for Clinical Reasoning Education. Int J Environ Res Public Health.

[32] Schubach F, Goos M, Fabry G, Vach W, Boeker M (2017). Virtual patients in the acquisition of clinical reasoning skills: does presentation mode matter? A quasi-randomized controlled trial. BMC Med Educ.

[33] Raupach T, Muenscher C, Anders S, Steinbach R, Pukrop T, Hege I, Tullius M (2009). Web-based collaborative training of clinical reasoning: a randomized trial. Med Teach.

[34] Plackett R, Kassianos AP, Kambouri M, Kay N, Mylan S, Hopwood J, Schartau P, Gray S, Timmis J, Bennett S (2020). Online patient simulation training to improve clinical reasoning: a feasibility randomised controlled trial. BMC Med Educ.

[35] Isaza-Restrepo A, Gomez MT, Cifuentes G, Arguello A (2018). The virtual patient as a learning tool: a mixed quantitative qualitative study. BMC Med Educ.

[36] Kahl K, Alte C, Sipos V, Kordon A, Hohagen F, Schweiger U (2010). A randomized study of iterative hypothesis testing in undergraduate psychiatric education. Acta Psychiatr Scand.

[37] Raupach T, de Insa T, Middeke A, Anders S, Morton C, Schuelper N (2021). Effectiveness of a serious game addressing guideline adherence: cohort study with 1.5-year follow-up. BMC Med Educ.

[38] Devitt P, Palmer E (1998). Computers in medical education 1: Evaluation of a problem-orientated learning package. Aust N Z J Surg.

[39] Qin YH (2022). Zixing; Yu, Jianqun; Qing, Ping; Lui, Su; Liu, Rongbo; Xiong, Jing; Wang, Peng; Lai, Yaning; Chen, Fan; Hu, Na: Practice-Based Learning Using Smart Class: A Competency-Based Model in Undergraduate Radiology Education. Acad Radiol.

[40] Lehmann R, Thiessen C, Frick B, Bosse HM, Nikendei C, Hoffmann GF, Tonshoff B, Huwendiek S (2015). Improving pediatric basic life support performance through blended learning with web-based virtual patients: Randomized controlled trial. J Med Internet Res.

[41] Wu B, Wang M, Johnson JM, Grotzer TA (2014). Improving the learning of clinical reasoning through computer-based cognitive representation. Med Educ Online.

[42] Bordage G, Page G (2018). The key-features approach to assess clinical decisions: validity evidence to date. Adv Health Sci Educ.

[43] Charlin B, Roy L, Brailovsky C, Goulet F, van der Vleuten C (2000). The Script Concordance Test: A Tool to Assess the Reflective Clinician. Teach Learn Med.

[44] Kalet AL, Coady SH, Hopkins MA, Hochberg MS, Riles TS (2007). Preliminary evaluation of the Web Initiative for Surgical Education (WISE-MD). Am J Surg.

[45] Bordage G, Grant J, Marsden P (1990). Quantitative assessment of diagnostic ability. Med Educ.

[46] Aghili O, Khamseh ME, Taghavinia M, Malek M, Emami Z, Baradaran HR, Mafinejad MK (2012). Virtual patient simulation: Promotion of clinical reasoning abilities of medical students. Knowl Manag E-Learn.

[47] Kleinert R, Heiermann N, Plum PS, Wahba R, Chang D-H, Maus M, Chon S-H, Hoelscher AH, Stippel DL (2015). Web-based immersive virtual patient simulators: Positive effect on clinical reasoning in medical education. J Med Internet Res.

[48] Botezatu M, Hult H, Tessma MK, Fors U (2010). Virtual patient simulation: Knowledge gain or knowledge loss?. Med Teach.

[49] Dekhtyar M, Park YS, Kalinyak J, Chudgar SM, Fedoriw KB, Johnson KJ, Knoche CF, Martinez L, Mingioni N, Pincavage AT, Salas R, Sanfilippo F, Sozio SM, Weigle N, Wood S, Zavodnick J, Stern S (2021). Use of a structured approach and virtual simulation practice to improve diagnostic reasoning. Diagnosis.

[50] Kalet AL, Song HS, Sarpel U, Schwartz R, Brenner J, Ark TK, Plass J (2012). Just enough, but not too much interactivity leads to better clinical skills performance after a computer assisted learning module. Med Teach.

[51] Mamede S, Schmidt HG (2004). The structure of reflective practice in medicine. Med Educ.

[52] Kassier J (1983). Teaching clinical medicine by iterative hypothesis testing. N Engl J Med.

[53] Homer BD, Plass JL (2014). Level of interactivity and executive functions as predictors of learning in computer-based chemistry simulations. Comput Hum Behav.

[54] Scott JN, Markert RJ, Dunn MM (1998). Critical thinking: change during medical school and relationship to performance in clinical clerkships. Med Educ.

[55] Niu L, Behar-Horenstein LS, Garvan CW (2013). Do instructional interventions influence college students’ critical thinking skills? A meta-analysis. Educ Res Rev.

[56] Viera AJ, Garrett JM (2005). Understanding interobserver agreement: the kappa statistic. Fam Med.

