# Supplementary material for: The effectiveness of using virtual patient educational tools to improve medical students’ clinical reasoning skills: a systematic review
Source: BMC Med Educ. 2022 May 13;22:365. doi: 10.1186/s12909-022-03410-x (PMC9098350; doi:10.1186/s12909-022-03410-x)
Supplement: Supplementary file 2 — Additional file 2. Search history for medline, embase, psychinfo. [file 12909_2022_3410_MOESM2_ESM.docx]

## Additional File 2:

**SEARCH HISTORY FOR MEDLINE, EMBASE, PSYCHINFO**

The search was modified for ERIC, CINAHL, Scopus and Web of Science

| 1. | Students, Medical/ |
| --- | --- |
| 2. | Education, Medical, Undergraduate/ |
| 3. | ((medic* adj3 student*) and undergraduate).mp. [mp=title, abstract, original title, name of substance word, subject heading word, keyword heading word, protocol supplementary concept word, rare disease supplementary concept word, unique identifier] |
| 4. | Computer-Assisted Instruction/ or computer assisted instruction.mp. [mp=title, abstract, original title, name of substance word, subject heading word, keyword heading word, protocol supplementary concept word, rare disease supplementary concept word, unique identifier] |
| 5. | Teaching/ |
| 6. | (online adj3 (learn* or educat* or teaching)).mp. [mp=title, abstract, original title, name of substance word, subject heading word, keyword heading word, protocol supplementary concept word, rare disease supplementary concept word, unique identifier] |
| 7. | exp Simulation Training/ |
| 8. | simulat*.mp. |
| 9. | virtual realit*.mp. |
| 10. | (patient* adj3 (virtual or simul*)).mp. [mp=title, abstract, original title, name of substance word, subject heading word, keyword heading word, protocol supplementary concept word, rare disease supplementary concept word, unique identifier] |
| 11. | (elearning or e-learning or electronic learning  or technology enhanced learning).mp. [mp=title, abstract, original title, name of substance word, subject heading word, keyword heading word, protocol supplementary concept word, rare disease supplementary concept word, unique identifier] |
| 12. | (learn* adj3 electron*).mp. [mp=title, abstract, original title, name of substance word, subject heading word, keyword heading word, protocol supplementary concept word, rare disease supplementary concept word, unique identifier] |
| 13. | 1 or 2 or 3 |
| 14. | 4 or 5 or 6 or 7 or 8 or 9 or 10 or 11 or 12 |
| 15. | Clinical Decision-Making/ |
| 16. | Decision Making/ |
| 17. | (making adj3 decision*).mp. [mp=title, abstract, original title, name of substance word, subject heading word, keyword heading word, protocol supplementary concept word, rare disease supplementary concept word, unique identifier] |
| 18. | decisionmaking.mp. |
| 19. | ((clinical or diagnostic) adj3 reasoning).mp. |
| 20. | clinical judg?ment.mp. |
| 21. | Judgment/ |
| 22. | (critical thinking or reasoning).mp. [mp=title, abstract, original title, name of substance word, subject heading word, keyword heading word, protocol supplementary concept word, rare disease supplementary concept word, unique identifier] |
| 23. | 15 or 16 or 17 or 18 or 19 or 20 or 21 or 22 |
| 24. | 13 and 14 and 23 |
| 25. | limit 24 to yr="1990 -Current" |
