# Supplementary material for: The effectiveness of using virtual patient educational tools to improve medical students’ clinical reasoning skills: a systematic review
Source: BMC Med Educ. 2022 May 13;22:365. doi: 10.1186/s12909-022-03410-x (PMC9098350; doi:10.1186/s12909-022-03410-x)
Supplement: Supplementary file 3 — Additional file 3. Quality assessment of included studies. [file 12909_2022_3410_MOESM3_ESM.docx]

Additional file 3 Quality assessment of included studies

| Authors | Theory informed evaluation (1)^[[1]](#footnote-1)^ | Validity and reliability of evaluation instrument (4)^[[2]](#footnote-2)^ | Sample from more than 1 medical school (1) | Representativeness of the Exposed Cohort sample (1)^[[3]](#footnote-3)^ | Selection of the Non-Exposed Cohort (1)^[[4]](#footnote-4)^ | Comparability of cohorts (2) ^[[5]](#footnote-5)^ | Blinding of outcome assessment (1) | Incomplete outcome data (1)^[[6]](#footnote-6)^ | Appropriate analysis (1) | Power calculation (1) | Total score (14) | Quality rating^[[7]](#footnote-7)^ |
| --- | --- | --- | --- | --- | --- | --- | --- | --- | --- | --- | --- | --- |
| Aghili et al. 2012 | 0 | 0 | 0 | 1 | 1 | 1 | 1 | 1 | 1 | 0 | 6 | Moderate |
| Botezatu et al. 2010 | 0 | 1 | 0 | 1 | 1 | 1 | 0 | 1 | 1 | 0 | 6 | Moderate |
| Chon et al. 2019 | 0 | 1 | 0 | 1 | 0 | 0 | 0 | 1 | 1 | 1 | 5 | Moderate |
| Dekhtyar et al. 2021 | 0 | 0 | 1 | 1 | 0 | 0 | 0 | 0 | 1 | 0 | 3 | Low |
| Devitt & Palmer 1998 | 0 | 0 | 0 | 1 | 1 | 2 | 0 | 1 | 1 | 0 | 6 | Moderate |
| Isaza-Restrepo et al. 2018 | 1 | 2 | 0 | 1 | 0 | 0 | 0 | 1 | 1 | 0 | 6 | Moderate |
| Kahl et al. 2010 | 1 | 1 | 0 | 1 | 1 | 1 | 0 | 1 | 1 | 0 | 7 | Moderate |
| Kalet et al. 2007 | 1 | 3 | 0 | 1 | 1 | 1 | 0 | 1 | 1 | 0 | 9 | Moderate |
| Kim et al. 2018 | 1 | 1 | 0 | 1 | 1 | 1 | 0 | 1 | 1 | 1 | 8 | Moderate |
| Kleinert et al. 2015 | 0 | 0 | 0 | 1 | 0 | 0 | 0 | 1 | 1 | 0 | 3 | Low |
| Lehmann et al. 2015 | 1 | 4 | 0 | 1 | 1 | 2 | 1 | 1 | 1 | 1 | 13 | High |
| Middeke et al 2018 | 1 | 0 | 0 | 1 | 1 | 1 | 0 | 1 | 1 | 0 | 6 | Moderate |
| Plackett et al 2020 | 1 | 3 | 1 | 1 | 1 | 2 | 0 | 0 | 1 | 1 | 11 | High |
| Qin et al. 2022 | 0 | 0 | 0 | 1 | 1 | 1 | 0 | 1 | 1 | 0 | 5 | Moderate |
| Raupach et al. 2009 | 1 | 3 | 0 | 1 | 1 | 1 | 0 | 1 | 1 | 1 | 10 | High |
| Raupach et al. 2021 | 0 | 1 | 0 | 1 | 1 | 1 | 0 | 0 | 1 | 0 | 5 | Moderate |
| Sobocan et al 2016 | 1 | 1 | 0 | 1 | 1 | 1 | 0 | 1 | 1 | 0 | 7 | Moderate |
| Watari et al 2020 | 0 | 0 | 0 | 1 | 0 | 0 | 0 | 1 | 1 | 0 | 3 | Low |
| Wu et al. 2014 | 1 | 0 | 0 | 1 | 0 | 0 | 1 | 1 | 1 | 0 | 5 | Moderate |

1. One point was given if the study described how theory informed the evaluation, by either describing theoretical frameworks they used to assess clinical reasoning or using previously developed and validated measures of clinical reasoning [↑](#footnote-ref-1)
2. One point was given if the study reported the inter-rater reliability, content validity, internal structure and relationships to other variables of the outcome variable. [↑](#footnote-ref-2)
3. One point was given if the study showed if the sample was representative of the average learner in this community. [↑](#footnote-ref-3)
4. One point was given if they showed the comparison group was drawn from the same community as the exposed cohort. [↑](#footnote-ref-4)
5. For randomised studies one point was given for randomisation and another for allocation concealment. For non-randomised studies one point was given if they controlled for baseline characteristics or outcome at baseline and two points were if awarded if they controlled for both baseline characteristics and the baseline outcome. [↑](#footnote-ref-5)
6. One point was given if ≥75% of participants were included at follow-up. [↑](#footnote-ref-6)
7. Scores of 0-4 equate to a low quality study, 5-9 moderate quality and 10-14 high quality. [↑](#footnote-ref-7)
